# Development of a Semi-Quantitative Food Frequency Questionnaire to Assess the Dietary Intake of a Multi-Ethnic Urban Asian Population

**DOI:** 10.3390/nu8090528

**Published:** 2016-08-27

**Authors:** Nithya Neelakantan, Clare Whitton, Sharna Seah, Hiromi Koh, Salome A. Rebello, Jia Yi Lim, Shiqi Chen, Mei Fen Chan, Ling Chew, Rob M. van Dam

**Affiliations:** 1 Saw Swee Hock School of Public Health, National University of Singapore and National University Health System, Singapore 117549; nithya.neelakantan@u.nus.edu (N.N.); clare_whitton@nuhs.edu.sg (C.W.); sharna.seah@u.nus.edu (S.S.); e0013172@u.nus.edu (H.K.); salome_antonette_rebello@nuhs.edu.sg (S.A.R.); 2 Research and Evaluation Department, Research and Strategic Planning Division, Health Promotion Board, Singapore 168937; LIM_Jia_Yi@hpb.gov.sg (J.Y.L.); CHEN_Shiqi@hpb.gov.sg (S.C.); Chan_Mei_Fen@hpb.gov.sg (M.F.C.); 3 Research and Strategic Planning Division, Health Promotion Board, Singapore 168937; chew_ling@hpb.gov.sg; 4 Department of Medicine, Yong Loo Lin School of Medicine, National University of Singapore and National University Health System, Singapore 117549; 5 Department of Nutrition, Harvard T.H. Chan School of Public Health, Boston, MA 02115, USA

**Keywords:** food frequency questionnaire, FFQ, development, methodology, multi-ethnic, dietary assessment, nutrition

## Abstract

Assessing habitual food consumption is challenging in multi-ethnic cosmopolitan settings. We systematically developed a semi-quantitative food frequency questionnaire (FFQ) in a multi-ethnic population in Singapore, using data from two 24-h dietary recalls from a nationally representative sample of 805 Singapore residents of Chinese, Malay and Indian ethnicity aged 18–79 years. Key steps included combining reported items on 24-h recalls into standardized food groups, developing a food list for the FFQ, pilot testing of different question formats, and cognitive interviews. Percentage contribution analysis and stepwise regression analysis were used to identify foods contributing cumulatively ≥90% to intakes and individually ≥1% to intake variance of key nutrients, for the total study population and for each ethnic group separately. Differences between ethnic groups were observed in proportions of consumers of certain foods (e.g., lentil stews, 1%–47%; and pork dishes, 0%–50%). The number of foods needed to explain variability in nutrient intakes differed substantially by ethnic groups and was substantially larger for the total population than for separate ethnic groups. A 163-item FFQ covered >95% of total population intake for all key nutrients. The methodological insights provided in this paper may be useful in developing similar FFQs in other multi-ethnic settings.

## 1. Introduction

Diet is an important modifiable risk factor for many diseases that are major contributors to morbidity and mortality worldwide [1]. Accurate information on dietary intakes is fundamental for various public health activities; for monitoring nutritional status, for prioritizing and assessing the impact of nutrition programs and interventions, and for identifying novel dietary risk factors for diseases [2]. Food frequency questionnaires (FFQ) are the most commonly used dietary instruments in epidemiological studies because they are more feasible to administer in large populations and are able to capture habitual dietary intake [3]. However, developing FFQs in the context of an increasingly globalized, cosmopolitan environment is challenging. Large diversity of food choices can lead to greater within-person and between-person variation in food intake and frequent out-of-home food consumption can limit knowledge of consumed food ingredients making recall more difficult. Dishes in Asian cuisines tend to be particularly complex containing multiple mixed ingredients that can be difficult to identify for persons that do not prepare the dishes. 

Another consideration for dietary assessment in cosmopolitan settings is how to address ethnic differences in food consumption. In some multi-ethnic populations, several key ethnic-specific food items were added to existing generic FFQs [4,5]; in such cases, the validity of the amended FFQ needs to be assessed. In other populations, multiple ethnic-specific FFQs were used [6], which may perform well within an ethnic group but could result in comparability issues across groups. As it is common for individuals to consume foods from other ethnic cuisines in cosmopolitan settings, an alternative approach is the use of a single FFQ developed for coverage of a multi-ethnic population as a whole [7,8,9]. While this approach is likely to be preferable in terms of comparability, achieving a high level of nutrient coverage could potentially result in a lengthy FFQ food list. 

Singapore is a multi-ethnic (74.3% Chinese, 13.3% Malay, 9.1% Indian and 3.2% others) [10], highly urbanized Asian country with a wide variety of traditional ethnic and international cuisines. Previously, an FFQ for Singaporeans was developed using food consumption data from 1993 (without ethnic group stratification) and was primarily designed for measuring intake of fatty acids and cholesterol, in order to better understand cardiovascular risk factors in the population [11]. Food consumption habits have changed in recent decades and there is an increasing interest in food components other than lipids, given the continuing rise in chronic disease prevalence [12]. We therefore used a data-driven approach to develop and pilot test a new comprehensive FFQ that accounts for ethnic-specific foods and mixed dishes with the use of data from two 24-h dietary recalls collected as part of the Singapore National Nutrition Survey. Few descriptions of the systematic development of FFQs in multi-ethnic settings are available in the literature [6,7,8,9,13]; therefore, our procedures and results can be useful for the development of FFQs in similar settings.

## 2. Materials and Methods 

### 2.1. 24-h Dietary Recall Data Collection

A 24-h dietary recall survey was conducted as part of the National Nutrition Survey 2010 in conjunction with the National Health Survey (NHS) 2010 in Singapore. In total, 1132 NHS-2010 participants aged 18–79 years were invited to take part in two 24-h dietary recalls and 805 participants provided their dietary recall for two intake days covering one weekday or working day and one weekend or non-working day within a month of each other between March and June 2010. Sampling was based on a selection matrix stratified by gender, ethnicity and age. Malay and Indian participants were over-sampled to provide adequate numbers for statistical comparisons between ethnic groups. Further details on the sampling methodology and sampling weights are published elsewhere [14]. 

Trained interviewers, using a multiple pass approach [15], conducted a face-to-face 24-h dietary recall interview at the participant’s household. Information regarding socio-demographic factors was also obtained during the interview. The 24-h dietary recall was used to obtain details of all foods and beverages consumed by the study participants over a 24-h period (midnight to midnight) of the day prior to the day of interview. Other information collected included time and venue of meal consumption, description of cooking methods and quantities of foods and beverages consumed. A compendium of food pictures and a set of standard household measures such as a bowl, plate, cup, glass and spoons of various sizes were used to aid the interview. Dietary data of the 24-h recalls were coded using the Food Information and Nutrient Database (FIND), Singapore, which housed Singapore food composition data [16]. The amount of energy and nutrients contributed by each food item was computed based on nutrient composition and weight. 

### 2.2. Development of the FFQ 

**Figure 1:**
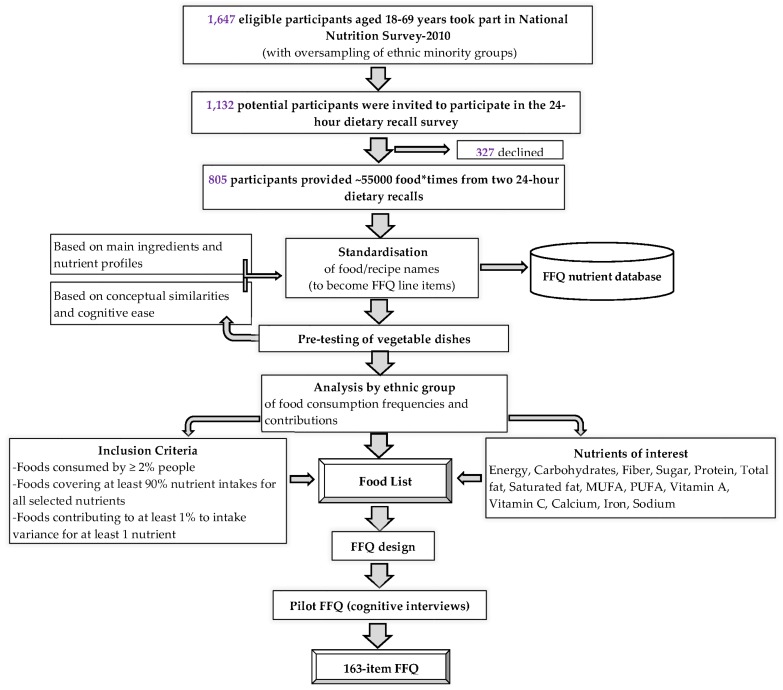
Flowchart of the food frequency questionnaire (FFQ) development study.

Using 24-h dietary recall data, a data-driven approach [3,17] was adopted to decide on the foods to be included in the FFQ. Cognitive interviews were used to inform decisions related to the wording and layout of the FFQ, portion size descriptions and response categories. Figure 1 illustrates the various steps involved in the development of the FFQ. Each step in Figure 1 is described below; some steps were iterative and informed previous ones.

#### 2.2.1. Standardization

We classified ~55,000 data points (i.e., consumption of various food items reported by study participants during the two 24-h dietary recall survey) into 272 food groups. To enable this classification, we standardized the individual food items (e.g., red/green apple with/without skin into one item “apple”) and assigned recipe names for each composite dish comprising multiple ingredients. Recipe names were standardized based on main ingredient (e.g., noodles) and cooking method (e.g., dry, fried, in gravy, and in soup), and similar recipes were grouped together (e.g., fishball noodle soup, and instant noodle soup). We created general food group names to classify food items under the broader food groups (e.g., “bread”, “rice dishes”, and “noodle dishes”). We retained the independent identity of staple foods and items reported by a large proportion of people (e.g., white rice, noodles, and roti prata). Ethnic rice preparations with different flavorings but with similar nutrient density were combined. Further, we split or excluded obscure or unidentifiable items from the composite dishes (e.g., bitter gourd with mayonnaise).

#### 2.2.2. Pre-Testing of Vegetables Dish Questions

Unlike meat and fish dishes where the main ingredient was generally clear, the variability of vegetables dishes was vast, often with multiple vegetables mixed together. It was therefore unclear whether to standardize vegetables (and dishes) as discrete items, subsequently asking for example, “How often do you consume cabbage?” or to standardize as groups of vegetables (and dishes) subsequently asking for example, “How often do you consume pale green leafy vegetables (cabbage/lettuce/cauliflower)?” To inform handling of single vs. mixed vegetable dishes, a pre-test of the FFQ vegetable section was conducted using six question formats in a convenience sample of 10 participants from various age, education, occupation and ethnic backgrounds. Participants consistently preferred single item vegetable lists and reported cognitive difficulties when vegetables were grouped due to differing consumption frequencies of each individual vegetable. Therefore, vegetable dishes were standardized into groups based on one main vegetable.

#### 2.2.3. Food List of the FFQ

In order to develop a food list for the FFQ, we focused on estimating total energy, carbohydrate, sugar, dietary fiber, protein, total fat, saturated fatty acid (SFA), mono-unsaturated fatty acid (MUFA), poly-unsaturated fatty acid (PUFA), calcium, vitamin A, vitamin C, iron and sodium. A separate food list was compiled for each of the three ethnic groups and then combined to ensure that the final FFQ adequately represented foods consumed by the main ethnic groups in Singapore. Foods consumed by ≥2% people, contributing cumulatively to ≥90% of key nutrient intakes, or explaining ≥1% of between-person intake variance were considered for inclusion in the food lists. These cut-offs were chosen because they were considered achievable based on other FFQ development studies in the literature [6,11,17,18], yet enough to achieve comprehensive coverage. In addition, foods of particular public health interest such as whole grains and berries were included. More details on these analyses are given in the Statistical Analyses section.

#### 2.2.4. FFQ Design

##### 2.2.4.1. Layout of the FFQ

Basic, familiar food items consumed by large proportions of the population, such as breads, were placed at the beginning of the questionnaire. Given their major contribution to nutrient intakes, staple foods (e.g., rice, noodles) were placed close to the start to avoid these items being subject to participant fatigue at the end or errors during initial familiarization at the start as suggested by Cade et al. (2002) [19]. Cognitively similar items were listed consecutively (e.g., different types of rice and rice dishes). FFQ layout was also informed by pre-testing and cognitive interviews.

##### 2.2.4.2. Sub-Type Questions

In order to reduce the length of the food list without losing key details on food intakes, we included sub-type questions, which could be skipped if the main food type was reportedly never consumed. We included questions on the cooking method (e.g., raw/steamed/boiled/stir-fried, etc. for vegetable dishes), type of oil used in food preparation and venue of obtaining food. Coffee, tea and malt beverages sections included sub-type questions for different types of milk and “less sugar” versions. We also asked sub-type questions on refined grains and whole grains. 

##### 2.2.4.3. Frequency of Food Consumption

Food frequency categories “per day/per week/per month/never or rarely” were used in which participants would be asked to provide a number of times within one of the categories. Since self-administered comprehensive FFQs were considered unsuitable for the population, we opted for this open-ended frequency category format for our interviewer-administered FFQ to allow for greater precision. We tested various response category wordings of sub-type questions in our cognitive interviews and we included the optimal format in the FFQ. For example, in one format the FFQ question “how often do you consume vegetables in curry with coconut?” used the response category wording, “never/rarely, sometimes, half the time, mostly, or always”.

##### 2.2.4.4. FFQ Portion Size

Standard portions were based on conceptually meaningful amounts (e.g., 1 bowl) and researcher judgment. Reference was also made to median portion sizes reported in the 24-h recall data. Local nutrition experts were consulted to assess the face validity of these portion sizes and portion size descriptors were tested during cognitive interviews.

### 2.3. Pre-Testing and Pilot Testing of the FFQ

Several strategies were adopted to test the FFQ and to identify potential difficulties in responding to FFQ questions. 

#### 2.3.1. Feedback from Local Food Experts and Stakeholders

Researchers familiar with local food habits checked the face validity of FFQ, appropriateness of portion sizes, incorrect description of foods and advised if any ethnic-specific local foods were missing from the FFQ food item examples. This included nutrition experts with experience in conducting the National Nutrition Survey, cohort studies, and nutrition interventions in Singapore. 

#### 2.3.2. Cognitive Interviews

Cognitive interviews were conducted to understand the thought processes the FFQ questions led to. Thirty participants (10 Chinese, 10 Malays, and 10 Indians) aged 25–75 years who were recruited via local newspaper advertisement or word-of-mouth. Using a standardized protocol which was developed using the verbal probing method [20], researchers administered the FFQ to participants probing on the following aspects: selection of food items (single food vs. mixed dish), estimation of mixed vegetable intake, information on food items, wording of food items (e.g., flavored rice), placement of food items, portion size, various formats of frequency of consumption, and FFQ layout. To ascertain how best to communicate standard serving sizes, participants were asked to compare various verbal descriptions and were also shown pictures of common household measurement tools such as dessert spoon, tea spoon, ladle, bowls, and cups. Each one-on-one cognitive interview took approximately 2 h. Written informed consent was obtained from all participants. This study was approved by the Institutional Review Board (reference code: B-14-082) at the National University of Singapore, Singapore. 

### 2.4. Nutrient Database

A nutrient database for the FFQ was constructed using the 24-h dietary recall data. Each food or beverage was tagged to an FFQ item ID and subsequently data were aggregated to generate a weighted nutrient profile for each FFQ item which reflected the relative consumption frequencies of each food sub-type included within each FFQ item.

### 2.5. Statistical Analysis

Descriptive statistics were used to describe socio-demographic characteristics of the study participants. Sampling weights were applied to the statistical analyses to account for unequal probability of selection and non-response rate. The weighted average intake of the two 24-h recalls was computed by summing 5/7ths of weekday intakes and 2/7ths of weekend intakes. After aggregation of all reported food items, the percentage of people consuming each of the 272 food items over the two-day period was calculated. Foods consumed by <2% people overall or within each ethnic group and contributing to intake of only one nutrient which could not be grouped elsewhere were excluded. Percentage contribution analysis was performed by calculating the contribution of each food item to total daily energy and selected key nutrients (carbohydrate, sugar, dietary fiber, protein, total fat, saturated fatty acid (SFA), mono-unsaturated fatty acid (MUFA), poly-unsaturated fatty acid (PUFA), calcium, vitamin A, vitamin C, iron and sodium). Foods that cumulatively contributed at least 90% to intakes were included in the food list. Foods that contributed at least 1% to intake variance were also considered for inclusion. Stepwise linear regression analyses were performed to assess the foods that explained at least 1% variance for each nutrient. This was done by modeling each key nutrient as a dependent variable and entering the 272 food items (grams per day) as independent variables. All analyses were conducted for the total study population and for each ethnic group separately. Statistical analyses were carried out in IBM SPSS version 23 (IBM Corp, Armonk, NY, USA) and the level of significance was set at 5%. 

## 3. Results

### 3.1. FFQ Development Phase

Table 1 shows characteristics of the 805 participants who took part in 24-h dietary recall survey for the total study sample and by ethnicity. The mean age was 44 (range: 18–79) years, 49.4% were females and the ethnic distribution was 39.5% Chinese, 30.3% Malay, and 30.2% Indians. The mean (standard deviation, SD) body mass index was 25.2 (4.2) kg/m^2^ for males and 25.3 (5.4) kg/m^2^ for females. In terms of age group distribution and educational and marital status, the sample reflected the general Singapore population [21].

Individual foods and mixed dishes reported in the dietary recall data were standardized into 272 food groups. The total number of food groups reported over the two-day dietary recall varied by ethnic group: Chinese, 207; Malay, 178; and Indian, 183. The key contributors to energy and nutrient intakes generally were food items consumed by large proportions of the population. White rice, fried noodles, poultry dishes, and white bread were commonly consumed in all ethnic groups and contributed to 13%–16%, 4%–7%, 4%–7% and 3%–5% of total energy intake, respectively (Table 2). 

Some food groups were consumed by smaller proportions of the population but made significant contributions to nutrient intakes because of high nutrient density, such as dhal (lentil stews) which was consumed by 47% of Indians and contributed to 9% of fiber intake, and puffs and pies which were consumed by 21% of Malays and contributed to 3% of energy intake. Although many food groups made important contributions to nutrient intakes in all ethnic groups such as white rice, white bread, poultry dishes, coffee and tea (Table 2), there were a number of foods for which contribution to nutrients, explanation of between-person variance (Table S1) and proportion of consumers varied greatly between ethnic groups. For example, the range of proportion of consumers across ethnic groups was 0%–25% for thosai (fermented rice pancake), 1%–47% for dhal, 0%–50% for pork dishes, 26%–47% for white fish dishes, 17%–38% for potato and 12%–37% for noodles in soup (Table 2). 

For the selection of foods for inclusion in the FFQ, we examined both their contribution to absolute energy and nutrient intakes and to variation in energy and nutrient intakes. Here we will focus on the results for fiber intake to illustrate our findings. The food items with the greatest contribution to fiber intakes were white rice, white bread, noodles, wholegrain products, dhal, fiber-rich vegetables, and fruits (Table S2). However, for both contribution to absolute intakes and variation in intakes the rankings of foods differed between the ethnic groups. For instance, in the Indian ethnic group, dhal was the biggest contributor (9%) to fiber intake and was the top food item explaining most of the variation in fiber intake (*R*^2^ = 0.26) in the regression model, but it was not a substantial contributor in the Chinese or Malay ethnic group due to low consumption. Table 3 shows similarities (e.g., noodles) as well as differences (e.g., idli, chapati, and dumpling) in the selection of foods that explained at least 60% of variability in fiber intake and contributions to absolute fiber intake across ethnic groups. In the dhal example above for Indians, both percentage contribution and regression analysis indicated that dhal should be included in the FFQ food list. This was the case for a number of foods while for other foods, the results from the two types of analyses indicated different items for the food list. For example, white rice contributed 5%–6% of total fiber intake, across ethnic groups. However, due to the limited between-person variability, white rice did not emerge as a substantial contributor to variability in fiber intake. Conversely, innards contributed to <1% of absolute fiber intake, but due to higher between-person variability it was among the top contributors to fiber intake among ethnic Indians.

**Figure 2:**
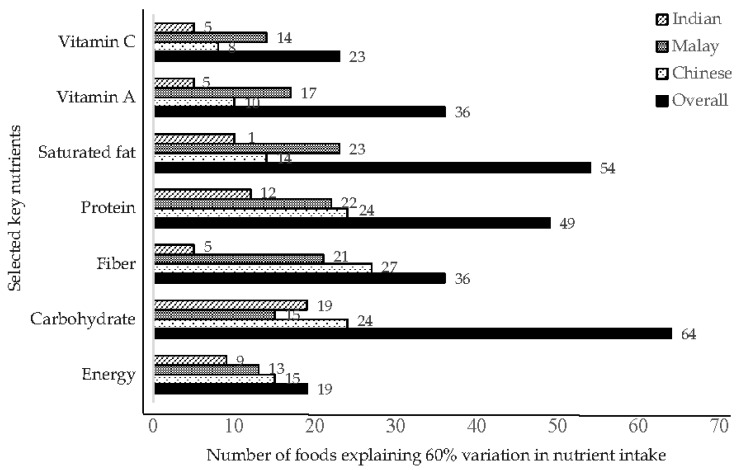
Number of foods that explained 60% of variation in energy, carbohydrate, fiber, protein, saturated fat, vitamin A and vitamin C intake in ethnic groups and the overall study population. Data are based on stepwise regression with nutrients as dependent and 272 standardized foods as independent variables.

Figure 2 presents the number of foods needed to explain at least 60% variability for the total energy and selected nutrients by ethnicity. For carbohydrates, the highest number of foods was needed to explain the between-person variability, whereas the smallest number of foods was required for vitamin A and C. For most nutrients measured, fewer foods were needed to explain the between-person variability among Indians as compared to the other ethnic groups. Due to the consumption of a large variety of foods, a relatively higher number of foods were needed to explain the variability among Chinese.

### 3.2. FFQ Testing Phase: Cognitive Interview Results

Most participants felt able to report within the specified timeframe of the past year by referring to habitual behaviors; (“If I work I will visit that stall usually three times per week. At times when I visit this hawker…I will always ask for the kangkong…so that’s how I remember”. male (M), 25 y, Malay); (“My pattern is the same…I can roughly say that” M, 40 y, Indian). Participants who had recently changed their diets were unsure how to answer, for example one participant had stopped consuming sugar-sweetened beverages 6 months previously. It was noted that interviewers would need to receive specific training on how to handle cases where intakes had changed over the past year.

The preferred wording for frequency categories for food sub-type questions (such as how often wholegrain varieties of a food were used) was “Never/Rarely”, “Sometimes”, “Half the time”, “Mostly”, and “Almost always or always”. 

There were varied opinions on the standard servings sizes used on the FFQ. For vegetables many participants easily identified with “scoop” and preferred this description to “cup” (“Nowadays at the stalls usually they straight away just serve you with that one scoop” female (F), 46 y, Malay). Describing meat as a “palm-sized piece” rather than a fillet/chop was preferred by most participants (“I can figure it out based on the hand”. F, 51 y, Chinese), but others felt it was easier to answer with reference to the scoop, especially when meat was cut into small pieces. 

Post-cognitive interview amendments to the FFQ included: changing the ordering of certain items, for example placing plain porridge before flavored to avoid double-counting; addition of examples such as for “flavored rice” for which the number of examples given increased from three to ten; and including multiple descriptions of serving sizes where appropriate, e.g., “1 fish fillet/1 palm-sized piece”.

### 3.3. Final FFQ 

The final food list comprised 163 items that contributed cumulatively to at least 95% of absolute intakes of energy and selected nutrients. There was little difference in the percentage of variance explained by the final food list compared with that explained by all standardized food groups. For example, the final food list accounted for 89.1% of between-person variation in energy intakes, while including all standardized food groups would account for 91.1%. For fiber the final food list accounted for 66.0% variation, while including all standardized food groups would account for 73.8%. The FFQ covered 18 sections reflecting different types of foods (e.g., bread, rice, noodles, etc.) with fixed serving sizes (e.g., 2 slices of bread, 1 rice bowl, and 1 plate of noodles) and four response categories to measure food frequency (per day, per week, per month, and never/rarely) over the past year. Administration time was approximately 45 min. The final FFQ is shown in Figure S1.

## 4. Discussion

We developed a food frequency questionnaire for adults of Indian, Malay, and Chinese ethnicity residing in Singapore, using data-driven methods with an emphasis on ensuring good coverage of foods and nutrients for each of the ethnic groups. Using national level 24-h recall data, we ascertained both overall and by ethnic group the proportion of people consuming each food, which foods were key contributors to absolute intakes of energy and nutrients, and which foods were responsible for a large proportion of between-person variation in nutrient intakes. Pre-tests, cognitive interviews and stakeholder input allowed us to refine the food list, wording and format of the questionnaire, resulting in a food list of 163 items which covers at least 95% of absolute intake and key sources of variability in intake of selected nutrients (energy, protein, carbohydrate, fat, MUFA, PUFA, SFA, sugar, fiber, vitamin A, vitamin C, iron, calcium and sodium), both in the general population and in major ethnic population sub-groups. Based on cognitive interviews, for all ethnic groups, the FFQ food item examples included items that appeared to be relevant and intuitive (for example, “flavored rice”, e.g., chicken rice, briyani, nasi lemak, pilau, nasi minyak, yellow rice, olive rice, tomato rice, saffron rice, and yam rice), and the question format appeared to be comprehendible at all levels of education. 

The variety of mixed dishes available in Singapore is wide, dishes generally have multiple minor ingredients, and eating out is common and as a result awareness of composition of dishes can be low. Therefore, the aim was for the FFQ to ask about these mixed dishes rather than the discrete items within a dish. In addition, the availability of recent population-level 24-h dietary recall data meant that an FFQ analysis database could be constructed containing nutrient profiles for each food list item that were weighted according to the relative consumption by the population of each variety of food within a food list item. The final food list comprises a number of mixed dishes, as well as dishes that have been minimally separated. More standard cereal-based mixed dishes such as noodle dishes remained as a whole dish on the FFQ. Rice-based dishes where the rice was mixed with meat/fish/vegetables before serving also remained as a whole dish. For dishes that comprised rice that was not mixed with meat/fish/vegetables, the components were asked about separately. For example, steamed rice, black pepper chicken, and kai-lan in oyster sauce would be captured in three different sections of the FFQ, i.e., “Rice”, “Poultry dishes” and “Vegetable dishes”, despite being consumed as one meal. While a possible consequence of having a combination of mixed and discrete items on the food list is that there is potential for items to be counted twice, it was thought that a food list of only discrete items would result in under-reporting because participants may not be aware of the contents and quantities of items within complex mixed dishes.

The nutrient coverage of our FFQ food groups was higher than our original cut-off of 90% because of the combination of ethnic-specific lists which each individually covered 90% of nutrient intakes. Nutrient coverage was similar to that observed in other studies using similar data-driven methods [6,17,18], while the food list was longer than most of these. Willet [3] recommends that an FFQ list should contain a maximum of 130 food items to limit participant burden. Our list was slightly longer because of the inclusion of ethnic-specific items, inclusion of discrete rather than grouped items (e.g., “spinach”, “kai-lan”, and “cabbage” rather than “green leafy vegetables”), and inclusion of individual food items of specific public health interest, such as whole grains and berries. However, participant burden is not only determined by the number of questions, but also by the amount of time taken to answer each question. Our FFQ contains a number of ethnic-specific items to which participants can immediately report non-consumption; for such questions the cognitive burden is minimal. Pre-testing of our questionnaire suggested that recalling intake frequency of grouped categories, such as “dark green leafy vegetables” was a greater cognitive challenge than to recall intake frequency of discrete items. This finding agrees with observations in previous studies [22]. Therefore, while our food list is relatively long, the burden and cognitive challenge of each individual question is relatively low compared to other FFQs. 

The number of food items required to explain the majority of between-person variance in our population was similar to observations in several studies [9,18,23] but much higher than reported in other studies [6,24,25,26,27,28]. It should be noted, however, that there are large methodological differences between these studies. Several studies evaluated the proportion of variance in nutrient intakes assessed with a longer FFQ that a new shorter FFQ is able to detect [25,27,28]. This approach is expected to lead to higher *R*^2^ values than our approach, because the number of food items in the longer FFQ is already limited as compared with the large number of food items in 24-h recalls. In our study, we modeled nutrient intakes from 24-h dietary recalls as the dependent variable, using potential FFQ food groups as the independent variables, because this most closely represents the level of detail that will be collected by the FFQ. Our findings are similar to those from several previous studies that also used nutrient data from open-ended dietary assessment methods with predictors as grams of potential FFQ food groups [9,18,23]. In a few other studies, a relatively short food list explained a higher proportion of variance than in our study [24,26], which may reflect a lower level of dietary diversity within these populations. 

Cognitive interviews led to refinements in frequency category wording, food group ordering, descriptions and examples. They also showed that there is no “one-size-fits-all” for certain FFQ aspects such as serving size descriptions. Rather than forcing participants to answer according to a measure that may not be relevant for them (e.g., palm-sized piece of chicken), we included two different but approximately equivalent standard servings sizes for a number of food items. For example, for fish, the standard serving is given as “1 fish fillet/1 palm-sized piece”. Although our cognitive interview sample size was modest, it was diverse in terms of age, gender and ethnicity. Many of the participants had some interest in food and nutrition, and had higher than average educational attainment. However, we did not observe that participants with low educational attainment had more or less cognitive difficulty with the FFQ as compared with participants with higher educational attainment. 

The aim was for the FFQ development to be data-driven and this was achieved to a large extent, but the process can never be completely objective. Standardization of foods/recipes into groups was conducted within a decision framework based on ingredients, nutrients and conceptual similarities supported by feedback from pre-tests and cognitive interviews. Creation of food groups, into which foods are standardized, however, is based on researchers’ judgment, for example, having a separate group for carbonated and non-carbonated soft drinks. Since the items on the final food list were based on cut-offs such as contributing to 90% of intake of at least one nutrient of interest, a different grouping strategy may have resulted in different food list items on the questionnaire. For example, items that were not covered in our final food list such as canned fruit and other grains would have been included if canned fruit had been grouped with fruit, and other grains had been grouped with rice at the initial standardization stage. This issue applies to all FFQs developed using data-driven methods.

Our FFQ was developed using two-day 24-h dietary recall data from a national sample of 805 participants. Using two days of dietary recall data rather than a single recall means that some intra-individual variation in food and nutrient intakes is accounted for, especially that related to day type (i.e., work day vs. non-work day). Although two days are unlikely to be enough to capture all of the food types eaten by an individual, this is compensated for by the size of the sample population. Thus values for percentage contribution of food to nutrient intakes should be fairly reliable. However, more caution is required when interpreting values such as proportion of consumers and between-person variation, which are more heavily affected by within-person variation. For these reasons, we believe that using both the contribution of foods to absolute energy and nutrient intakes and variability in these intakes is valuable. The relative validity of our new FFQ in comparison to other dietary assessment methods is currently being tested. The 24-h dietary recall data used also had some potential limitations. Firstly, the data were collected in 2010 but new food products and trends emerge regularly, so it is possible that certain important items are missing from the food list simply because they did not appear in the recall data and it was decided not to add items to the list based on anecdotal evidence. The two days of dietary recall were mostly collected within two weeks of each other, between March and June 2010. While seasonal variation was not well captured in this data, seasonal variation in Singapore is mostly limited to some fruits and foods associated with cultural/religious festivals, such as mooncakes and pineapple tarts. This was accounted for in part by adding common seasonal foods to the FFQ such as durian. Foods consumed at specific festivals may not be well captured, but the impact of these items on habitual intakes would be low. Some under-reporting in 24-h recalls is common due to incomplete recall of foods and desirability bias, but such under-reporting would probably have to be common and severe to lead to missing key foods from the FFQ list. Another limitation is that for sugar, but not for other nutrients, the food composition database contained missing values (12% of the food codes of which around 20% plausibly contain substantial amounts of sugar). Most of the local dishes in the food composition data had nutrient profiles derived from laboratory analysis of a number of samples purchased from food outlets, which is a major strength of the 24-h dietary recall data, and provides confidence in the nutrient coverage estimates. 

## 5. Conclusions

In the present study, an FFQ for the adult multi-ethnic Singapore population was developed with good coverage of foods and beverages contributing to intakes of energy, carbohydrate, protein, fat, SFA, MUFA, PUFA, sugar, fiber, vitamin A, vitamin C, calcium, iron and sodium. The intention is for this FFQ to be used in future surveys, for epidemiology and surveillance purposes, replacing the local FFQ developed in the 1990s. This should allow for more robust insights into diet-disease associations for a range of nutrients. An on-going validation study including repeat 24-h dietary recalls, collection of biomarkers and repeat administrations of the FFQ to assess reproducibility will provide insights into the performance of the FFQ. We have described our approach to developing this FFQ and associated nutrient analysis database, suitable for a diverse multi-ethnic population, and have outlined the challenges faced and solutions adopted. The experience of developing the FFQ provided us with several key lessons. Firstly, the percentage contribution and stepwise regression analyses were supplementary in that they highlighted different foods for inclusion in the food list. Secondly, a combination of mixed dishes and more discrete items were required on the FFQ for reporting ease and better accuracy, and this may be applicable to other Asian settings. Thirdly, in order to balance the length of the questionnaire with coverage, broad researcher-defined categories were sometimes required, which may not be instantly familiar to participants. Therefore, several examples for each line item were often required to ensure variants relevant to every ethnic group were covered. In an increasingly globalized world, with widespread availability of international food choices, such methodological insights are relevant in a growing number of cosmopolitan settings, and the resulting dietary assessment tools will be instrumental for research to explain the role of ethnicity in diet-disease relationships.

## Figures and Tables

**Table 1:** Characteristics of the participants of the 24-h dietary recall survey.

Characteristics	Total Sample	Chinese	Malay	Indian	*p*-Value
	(*n* = 805)	(*n* = 318)	(*n* = 244)	(*n* = 243)	
Age (years)	44.5 ± 16.0	44.5 ± 16.4	43.9 ± 16.1	45.0 ± 15.5	0.72
Females, *n* (%)	398 (49.4)	161 (50.6)	122 (50.0)	115 (47.3)	0.72
Educational status, *n* (%)					
Primary education or less	161 (20.0)	56 (17.6)	54 (22.1)	51 (21.1)	
Secondary school	309 (38.4)	109 (34.3)	124 (50.8)	76 (31.4)	<0.001
Higher education including vocational	198 (24.6)	79 (24.8)	59 (24.2)	60 (24.8)	
University	135 (16.8)	74 (23.3)	6 (2.5)	55 (22.7)	
Marital status, *n* (%)					
Single	173 (21.5)	80 (25.2)	51 (20.9)	42 (17.4)	
Married	555 (69.0)	213 (67.0)	172 (70.5)	170 (70.2)	0.11
Others ^†^	76 (9.5)	25 (7.9)	21 (8.6)	30 (12.4)	
Working status, *n* (%)					
Employed	519 (64.6)	207 (65.1)	155 (63.5)	157 (64.9)	
Homemaker	144 (17.9)	49 (15.4)	44 (18.0)	51 (21.1)	
Retired	64 (8.0)	26 (8.2)	24 (9.8)	14 (5.8)	0.002
Unemployed	17 (2.1)	1 (0.3)	6 (2.5)	10 (4.1)	
Student/National service	60 (7.5)	35 (11.0)	15 (6.1)	10 (4.1)	
Family income (S$ per month), *n* (%)					
<2000	174 (21.6)	47 (14.8)	75 (30.7)	52 (21.5)	
2000–3999	222 (27.6)	67 (21.1)	78 (32.0)	77 (31.8)	
4000–5999	157 (19.5)	67 (21.1)	43 (17.6)	47 (19.4)	<0.001
≥6000	138 (17.1)	79 (24.8)	17 (6.9)	42 (17.4)	
Don’t know/refused	113 (14.1)	58 (18.2)	31 (12.7)	24 (9.9)	
Body mass index (kg/m^2^)	25.2 ± 4.9	23.6 ± 3.9	26.6 ± 5.6	26.1 ± 4.6	<0.001

Values are means ± SDs for continuous variables; number and percentages for categorical variable; ^†^ separated, divorced and widowed; S$, Singapore dollars.

**Table 2:** Top 20 contributors according to percent contribution to total energy intake in the 24-h dietary recall study by ethnicity.

	Chinese (*n* = 318)			Malay (*n* = 244)			Indian (*n* = 243)		
Rank	Food items	Energy (%)	% consumers	Food items	Energy (%)	% consumers	Food items	Energy (%)	% consumers
1	White rice	12.7	81.1	White rice	15.9	88.1	White rice	16.0	85.6
2	Fried noodles	6.8	42.1	Chicken dishes	7.3	64.3	White bread	4.9	52.7
3	Noodles in soup	6.3	37.1	White bread	4.7	54.5	Fried noodles	4.4	30.0
4	Chicken dishes	4.6	60.1	Fried noodles	4.3	30.3	Chicken dishes	3.8	43.6
5	Pork dishes	3.9	50.3	White fish dishes	3.9	44.3	Coffee	3.5	62.1
6	Dry noodles	3.2	22.0	Flavored rice	3.4	33.6	Thosai ^3^	2.7	25.1
7	White fish dishes	3.0	46.5	Oily fish dishes	3.0	43.0	Flavored rice	2.7	23.5
8	White bread	2.7	42.5	Coffee	2.9	57.4	Dhal ^4^	2.3	46.5
9	Coffee	2.4	56.9	Puffs and pies	2.8	20.9	Chapati ^5^	2.3	17.3
10	Flavored rice	2.3	22.6	Tea	2.0	58.6	White fish dishes	2.0	26.3
11	Fried rice	1.6	11.0	Beef dishes	2.0	24.6	Noodles in soup	1.9	11.5
12	Oily fish dishes	1.5	28.6	Non-carbonated sweetened drinks ^1^	1.9	31.1	Wholemeal bread	1.8	23.0
13	Nuts	1.4	4.7	Noodles in soup	1.6	15.2	Tea	1.8	51.0
14	Filled buns, savory	1.2	12.6	Roti prata ^2^	1.5	19.7	Oily fish dishes	1.6	23.9
15	Dumplings	1.2	13.5	Fried rice	1.5	10.2	Mutton and lamb dishes	1.4	11.9
16	Non-carbonated sweetened drinks ^1^	1.1	29.2	Malted drinks	1.5	29.9	Potato dishes	1.4	37.9
17	Tea	1.1	44.0	Noodles in gravy	1.3	10.2	Roti prata	1.4	16.5
18	Soybean curd dishes	1.1	28.3	Fried chips and crackers, savory	1.2	20.5	Malted drinks	1.3	23.9
19	Flavored white rice porridge	1.1	12.9	Processed chicken products	1.2	15.6	Soybean curd dishes	1.3	18.1
20	Red/brown rice	1.1	7.5	Mutton and lamb dishes	1.2	11.5	Non-carbonated sweetened drinks	1.3	18.5

^1^ excluding juices and bottled tea/coffee; ^2^ flour-based pancake; ^3^ fermented rice pancake/crepe; ^4^ lentil stew; ^5^ whole wheat Indian bread.

**Table 3:** Foods that explained at least 60% of variation in total fiber intake and their contribution to total fiber intake in the 24-h dietary recall survey by ethnicity.

	Chinese (*n* = 318)			Malay (*n* = 244)			Indian (*n* = 243)		
Rank	Food items	Cum *R*^2 †^	% of fiber intake ^‡^	Food items	Cum *R*^2 †^	% of fiber intake ^‡^	Food items	Cum *R*^2 †^	% of fiber intake ^‡^
1	Pears	0.11	2.78	Lontong dishes ^5^	0.09	1.30	Dhal	0.26	9.22
2	Chocolate	0.16	0.55	Chicken dishes	0.16	6.04	Innards	0.39	0.00
3	Fried noodles	0.20	7.05	Puffs and pies	0.22	2.77	Chapati ^7^	0.46	3.25
4	Ice cream ^1^	0.24	0.49	Roti prata ^6^	0.26	1.76	Other grains ^8^	0.54	0.43
5	Sweet desserts in soup	0.26	1.32	Dumplings	0.30	0.58	Idli ^9^	0.61	1.62
6	Egg, fried/scrambled/braised	0.29	0.02	Egg, fried/scrambled/braised	0.33	0.03	Red/brown rice	0.64	0.69
7	French fries	0.32	1.17	Flavored rice	0.36	3.69	Pasta with meat/fish/vegetables	0.67	0.45
8	Red/brown rice	0.35	1.30	Pancake/hotcake/waffle	0.38	0.88	Flavored wholegrain breakfast cereal	0.70	0.02
9	Nuts	0.37	1.58	Oranges	0.40	0.63	Nuts	0.72	1.11
10	Olives	0.38	0.01	Fried chips and crackers, savory	0.43	1.20	Papaya	0.74	1.69
11	Papaya	0.40	1.72	Beef dishes	0.45	1.93	Soya milk, low sugar	0.75	0.20
12	Lettuce	0.42	0.12	Bean dishes	0.47	1.33	Bean dishes	0.77	1.58
13	Fried rice with vegetables	0.44	0.39	Milkshake	0.49	0.20	Ice cream ^1^	0.78	0.27
14	Noodles in soup	0.45	5.29	Cauliflower dishes	0.50	0.22	Savory fried snack	0.79	0.79
15	100% fruit or vegetable juice	0.47	0.44	Fried noodles	0.52	4.25	Chicken dishes	0.79	2.12
16	Flavored rice	0.48	3.09	Wholemeal bread	0.53	2.25	Filled buns, sweet	0.80	0.71
17	Oranges	0.49	1.07	Sweet desserts in soup (with coconut)	0.55	0.61	Guava	0.81	0.73
18	Potato dishes	0.51	1.38	Powdered nutrition drink	0.57	0.00	Wholemeal bread	0.81	4.10
19	Dragon fruit	0.52	0.57	White fish dishes	0.58	3.29	Fried noodles	0.82	4.18
20	Pasta with meat/fish/vegetables	0.53	0.90	Fried rice, plain or with meat/fish	0.59	0.85	Avocado	0.83	0.40
21	Non-carbonated sweetened drinks ^2^	0.54	0.28	Mutton and lamb dishes	0.60	0.89	Noodles in soup	0.83	1.25
22	Filled buns, savory	0.55	1.63	Peanut butter	0.61	0.49	Plain wholegrain breakfast cereal	0.84	0.16
23	Lotus root dishes	0.57	0.13	Pears	0.62	0.93	Sweet desserts in soup	0.84	1.00
24	Kaya ^3^	0.58	0.06	Whole milk	0.63	0.01	Apples	0.85	2.69
25	Dhal ^4^	0.58	0.21	Soya milk, low sugar	0.64	0.29	Dry noodles	0.85	0.38
26	Gourd dishes	0.59	0.53	Preserved vegetables	0.64	0.05	Cured pork products	0.86	0.05
27	Canned meat	0.60	0.03	Cabbage dishes	0.65	1.50	Banana flower	0.86	0.00

^†^ Cumulative *R*^2^ of the stepwise regression model. The ordering shows the process and in the final model, values may no longer be ordered according to size since addition of later items may reduce or increase values of earlier items; ^‡^ percent contribution to total fiber intake; ^1^ Ice cream with or without toppings; ^2^ excluding juices and bottled tea/coffee; ^3^ coconut jam; ^4^ lentil stew; ^5^ compressed rice cake; ^6^ flour based pancake; ^7^ whole wheat Indian bread; ^8^ e.g., semolina, and millet; ^9^ savory steamed rice cake.

## Supplementary Materials

The following are available online at http://www.mdpi.com/2072-6643/8/9/528/s1, Table S1: Foods that explained at least 60% of between-person variation in total energy intake in the 24-h dietary recall survey by ethnicity; Table S2: Top 20 contributors according to percent contribution to total fiber intake in the 24-h dietary recall study by ethnicity; Figure S1: The food frequency questionnaire

## Abbreviations

The following abbreviations are used in this manuscript:

F	female
FFQ	food frequency questionnaire
FIND	food information and nutrient database
M	male
MUFA	mono-unsaturated fatty acid
NHS	national health survey
PUFA	poly-unsaturated fatty acid
SD	standard deviation
SFA	saturated fatty acid
y	years
