# Supplementary material for: Development of a Semi-Quantitative Food Frequency Questionnaire to Assess the Dietary Intake of a Multi-Ethnic Urban Asian Population
Source: Nutrients. 2016 Aug 27;8(9):528. doi: 10.3390/nu8090528 (PMC5037515; doi:10.3390/nu8090528)
Supplement: Supplementary file 1 [file nutrients-08-00528-s001.zip › nutrients-142106-supplementary-publish/Suppl_materials/Figure S1_Long FFQ.pdf]

Last updated 02/12/2015

Study ID__________

Date_____________

Food Frequency Questionnaire

This questionnaire asks about foods eaten over the past year.
Participants responses should be the number of times either 'per day', 'per week', or 'per month'.

BREAD AND BREAD SPREADS

How often do you consume one serving of:

		Per day	Per week	Per month	Never/Rarely	*One serving means:*
1	**White bread** (inc. naan)				☐	*2 slices*
2	**Wholemeal bread**				☐	*2 slices*
3	**Bread with dried fruit/nuts/seeds** e.g. walnut bread, raisin bread, corn bread, multigrain				☐	*2 slices*
4	**Margarine** e.g. olive spread, Planta®				☐	*1 teaspoon*
5	**Butter**				☐	*1 teaspoon*
6	**Kaya**				☐	*1 teaspoon*
7	**Peanut butter**				☐	*1 teaspoon*
8	**Jam/Marmalade/Honey**				☐	*1 teaspoon*
9	**Chocolate spread** e.g. Nutella®				☐	*1 teaspoon*
10	**French toast**				☐	*2 triangular slices*
11	**Pizza**				☐	*1 slice (from 14" pizza)*
12	**Thosai, plain or with filling** e.g. potato				☐	*1 piece*
13	**Roti prata, plain or with egg/cheese** (inc. paratha)				☐	*1 piece*
14	**Chapati**				☐	*1 piece*
15	**Murtabak**				☐	*¼ of a whole/ 2 small pieces*
16	**Puri** (deep-fried Indian bread)				☐	*1 piece*
17	**Sweet filled buns** (inc. pau) e.g. red bean, coconut, custard				☐	*1 piece*
18	**Savoury filled buns** (inc. pau) e.g. chicken, char siew, cheese, hot dog				☐	*1 piece*

RICE

How often do you consume one serving of:

		Per day	Per week	Per month	Never/Rarely	One serving means:
19	**Rice, plain, boiled/steamed** (excluding porridge)				☐	*1 rice bowl*

How often is the rice:

		Never/Rarely	Sometimes	Half the time	Mostly	Always
19a	**Mixture of white and brown/red**	☐	☐	☐	☐	☐
19b	**Brown or red rice (unpolished)**	☐	☐	☐	☐	☐
19c	**White rice** (inc. basmati)	☐	☐	☐	☐	☐

How often do you consume one serving of:

		Per day	Per week	Per month	Never/Rarely	One serving means:
20	**Rice porridge, flavoured, (exc. plain)** e.g. chicken, pork, duck, fish, vegetarian				☐	*1 medium bowl/ 1 soup bowl(half full)*

How often is the porridge made with:

		Never/Rarely	Sometimes	Half the time	Mostly	Always
20a	**Mixture of white and brown/red**	☐	☐	☐	☐	☐
20b	**Brown or red rice (unpolished)**	☐	☐	☐	☐	☐
20c	**White rice**	☐	☐	☐	☐	☐

How often do you consume one serving of:

		Per day	Per week	Per month	Never/Rarely	One serving means:
21	**Rice porridge, plain**				☐	*1 medium bowl*

How often is the porridge made with:

		Never/Rarely	Sometimes	Half the time	Mostly	Always
21a	**Mixture of white and brown/red**	☐	☐	☐	☐	☐
21b	**Brown or red rice (unpolished)**	☐	☐	☐	☐	☐
21c	**White rice**	☐	☐	☐	☐	☐

How often do you consume one serving of:

		Per day	Per week	Per month	Never/Rarely	*One serving means:*
**22**	**Fried rice**				☐	*1 plate*

How often is the fried rice with:

		Never/Rarely	Sometimes	Half the time	Mostly	Always
**22a**	**With vegetables**	☐	☐	☐	☐	☐

How often is the fried rice made with:

		Never/Rarely	Sometimes	Half the time	Mostly	Always
**22b**	**Mixture of white and brown/red**	☐	☐	☐	☐	☐
**22c**	**Brown or red rice (unpolished)**	☐	☐	☐	☐	☐
**22d**	**White rice**	☐	☐	☐	☐	☐

How often is the fried rice prepared:

		Never/Rarely	Sometimes	Half the time	Mostly	Always
**22e**	**At home**	☐	☐	☐	☐	☐
**22f**	**By food outlet**	☐	☐	☐	☐	☐

How often do you consume one serving of:

		Per day	Per week	Per month	Never/Rarely	*One serving means:*
**23**	**Flavoured rice**<br>Chicken rice, briyani, nasi lemak, pilau, nasi minyak, yellow rice, olive rice, tomato rice, saffron rice, yam rice				☐	*1 rice bowl*
**24**	**Flavoured rice dish (mixed with meat/veg)** e.g. bi bim bap, claypot, mui fan, fan choy				☐	*1 medium bowl*
**25**	**Glutinous rice**<br>e.g. lor mai kai, bak chang				☐	*1 rice bowl,*<br>*1 piece*
**26**	**Lontong**				☐	*4 pieces*
**27**	**Idli (steamed rice cake, savoury)**				☐	*2 pieces*
**28**	**Rice roll**<br>e.g chwee kway, chee cheong fun				☐	*1 piece*

GRAVY

How often do you consume one serving of:

		Per day	Per week	Per month	Never/Rarely	One serving means:
**29**	**Gravy, with coconut (<u>extra</u> on top of rice or as a side dish)** e.g. curry gravy				☐	*1 scoop*
**30**	**Gravy, without coconut (<u>extra</u> on top of rice or as a side dish)** e.g. braised gravy, sambhar				☐	*1 scoop*

NOODLES

How often do you consume one serving of:

		Per day	Per week	Per month	Never/Rarely	One serving means:
**31**	**Noodles, fried** e.g. mee goreng, Hokkien mee, hor fun				☐	*1 plate*
**32**	**Noodles, in gravy** e.g laksa, mee rebus, mee siam, lor mee, curry noodles				☐	*1 medium bowl*
**33**	**Noodles, in soup** e.g. yong tau foo, mee soto, ramen, instant noodle soup, fishball noodles, sliced fish bee hoon				☐	*1 medium bowl*
**34**	**Noodles, dry** e.g. instant, dry wanton noodles, fishball noodles, mincemeat and mushroom noodles				☐	*1 medium bowl*

How often are the noodles:

		Never/Rarely	Sometimes	Half the time	Mostly	Always
**34a**	**White rice noodles** e.g. bee hoon, kway teow, mee tai mak	☐	☐	☐	☐	☐
**34b**	**Brown rice noodles** e.g. brown rice bee hoon	☐	☐	☐	☐	☐
**34c**	**Wheat noodles** e.g. yellow noodles, ban mian, mee hoon kueh, you-mian, sheng mien, yee mien, mee pok, mee kia, mee sua, ramen, soba, udon	☐	☐	☐	☐	☐
**34d**	**Instant noodles**	☐	☐	☐	☐	☐

How often are the noodles prepared:

		Never/Rarely	Sometimes	Half the time	Mostly	Always
**34e**	**At home**	☐	☐	☐	☐	☐
**34f**	**By food outlet**	☐	☐	☐	☐	☐

PASTA

How often do you consume one serving of:

		Per day	Per week	Per month	Never/Rarely	*One serving means:*
35	**Pasta, plain (without meat/fish/seafood/veg)** e.g. aglio olio				☐	*1 plate*
36	**Pasta, with meat/fish/seafood/veg**				☐	*1 plate*

How often is the pasta:

		Never/Rarely	Sometimes	Half the time	Mostly	Always
36a	**Wholemeal** (browner colour, rough texture)	☐	☐	☐	☐	☐

How often is the pasta made with:

		Never/Rarely	Sometimes	Half the time	Mostly	Always
36b	**Tomato sauce**	☐	☐	☐	☐	☐
36c	**Creamy sauce** e.g. cheese sauce	☐	☐	☐	☐	☐
36d	**Without any sauce**	☐	☐	☐	☐	☐

FRENCH FRIES

How often do you consume one serving of:

		Per day	Per week	Per month	Never/Rarely	*One serving means:*
37	**French fries** (inc. wedges, crinkle cut)				☐	*1 regular serving*

BREAKFAST CEREAL

How often do you consume one serving of:

		Per day	Per week	Per month	Never/Rarely	*One serving means:*
38	**Oats** e.g. rolled oats, instant				☐	*1 medium bowl*
39	**3-in-1 cereal** e.g. Nutremill®, Nestum®				☐	*1 cup*
40	**Other breakfast cereal** **PLEASE STATE BRAND AND TYPE**					
40a	________________________________				☐	*1 medium bowl (before adding milk)*
40b	________________________________				☐	*1 medium bowl (before adding milk)*

POULTRY DISHES

How often do you consume one serving of:

		Per day	Per week	Per month	Never/Rarely	One serving means:
**41**	**Chicken/turkey ham**					*4 slices ham*
**42**	**Processed chicken products** e.g. nugget, sausage, burger, hot dog				☐	*6 nuggets; 1 sausage/burger*
**43**	**Chicken/duck, fresh (not processed)** Including chicken from mixed dishes such as chicken rice, nasi lemak				☐	*1 palm-sized piece*

		Never/Rarely	Sometimes	Half the time	Mostly	Always
**43a**	**How often do you remove the skin from chicken/duck:**	☐	☐	☐	☐	☐
	How often do you consume chicken/duck:					
**43b**	**In curry with coconut**	☐	☐	☐	☐	☐
**43c**	**In curry without coconut**	☐	☐	☐	☐	☐
**43d**	**Stir-fried/pan-fried**	☐	☐	☐	☐	☐
**43e**	**Deep-fried**	☐	☐	☐	☐	☐
**43f**	**Stewed/braised**	☐	☐	☐	☐	☐
**43g**	**Roasted/grilled/baked**	☐	☐	☐	☐	☐
**43h**	**Boiled/steamed/in soup**	☐	☐	☐	☐	☐
	How often do you consume chicken/duck prepared:					
**43i**	**At home**	☐	☐	☐	☐	☐
**43j**	**By food outlet**	☐	☐	☐	☐	☐

FISH AND SEAFOOD DISHES

How often do you consume one serving of:

		Per day	Per week	Per month	Never/Rarely	One serving means:
**44**	**Raw fish/sashimi (no rice)**				☐	*1 piece*
**45**	**Sushi roll/nigiri (includes rice)**				☐	*3 pieces, 1 handroll*
**46**	**Salted fish/dried fish** e.g. ikan bilis				☐	*1 dessertspoon*
**47**	**Canned fish** e.g. tuna, sardines, dace				☐	*1 can (150g)*

Excluding the fish above, how often do you consume one serving of:

		Per day	Per week	Per month	Never/Rarely	One serving means:
**48**	**White fish** (dry and flaky)<br>e.g. pomfret, snapper, bream, catfish, grouper, sea bass, stingray, kuning, cod, dory, fish ball, fish cake				☐	*1 fish fillet/<br>1 palm-sized piece*
**49**	**Oily/fatty fish**<br>(solid texture, darker colour)<br>eg. mackerel/kembong/batang/saba, yellowtail trevally, salmon, sardine, tuna				☐	*1 fish fillet/<br>1 palm-sized piece*
**50**	**Prawns**				☐	*1 scoop/<br>4 medium prawns*
**51**	**Crab**				☐	*half a small crab*
**52**	**Squid/sotong, cuttlefish, octopus, abalone**				☐	*1 scoop*
**53**	**Oysters/cockles/mussels/scallops**				☐	*1 scoop<br>(without shells)*

How often do you consume fish/seafood (e.g. fish, prawns, crab, sotong):

		Never/Rarely	Sometimes	Half the time	Mostly	Always
**53a**	**In curry with coconut**	☐	☐	☐	☐	☐
**53b**	**In curry without coconut**<br>e.g. asam pedas, asam rebus	☐	☐	☐	☐	☐
**53c**	**Stir-fried/pan-fried**	☐	☐	☐	☐	☐
**53d**	**Deep-fried**	☐	☐	☐	☐	☐
**53e**	**Stewed/braised**	☐	☐	☐	☐	☐
**53f**	**Roasted/grilled/baked**	☐	☐	☐	☐	☐
**53g**	**Boiled/steamed/in soup**	☐	☐	☐	☐	☐

How often do you consume fish/seafood prepared:

		Never/Rarely	Sometimes	Half the time	Mostly	Always
**53h**	**At home**	☐	☐	☐	☐	☐
**53i**	**By food outlet**	☐	☐	☐	☐	☐

MEAT DISHES

How often do you consume one serving of:

		Per day	Per week	Per month	Never/Rarely	*One serving means:*
54	**Organ meat (spare parts)** e.g. liver, paru/lung, kidney, intestines				☐	*1 scoop*
55	**Cured pork products** e.g. bacon, ham, lap cheong, luncheon meat				☐	*1 sausage, 2 slices bacon/ham; 1 slice luncheon meat*
56	**Pork, fresh**				☐	*1 palm-sized piece*
57	**Beef burger** (inc. bread bun)				☐	*1 burger*
58	**Beef, fresh** (excluding burgers)				☐	*1 palm-sized piece*
59	**Mutton/Lamb**				☐	*1 palm-sized piece*

		Never/Rarely	Sometimes	Half the time	Mostly	Always
**59a**	**How often is there visible fat on the meat you consume?**	☐	☐	☐	☐	☐
	How often do you consume meat (e.g. beef, pork, mutton):					
**59b**	**In curry with coconut**	☐	☐	☐	☐	☐
**59c**	**In curry without coconut**	☐	☐	☐	☐	☐
**59d**	**Stir-fried/pan-fried**	☐	☐	☐	☐	☐
**59e**	**Deep-fried**	☐	☐	☐	☐	☐
**59f**	**Stewed/braised**	☐	☐	☐	☐	☐
**59g**	**Roasted/grilled/baked**	☐	☐	☐	☐	☐
**59h**	**Boiled/steamed/in soup**	☐	☐	☐	☐	☐
	How often do you consume meat prepared:					
**59i**	**At home**	☐	☐	☐	☐	☐
**59j**	**By food outlet**	☐	☐	☐	☐	☐

EGG AND EGG DISHES

How often do you consume one serving of:

Egg-based mixed dishes (egg is the main ingredient)
e.g. omelette, steamed egg, scrambled egg with vegetables, chawanmushi

		Per day	Per week	Per month	Never/Rarely	One serving means:
**60**	containing **Pork**				☐	*1 scoop/1 piece*
**61**	containing **Chicken**				☐	*1 scoop/1 piece*
**62**	containing **Fish**				☐	*1 scoop/1 piece*
**63**	containing **Beancurd**				☐	*1 scoop/1 piece*
**64**	containing **Vegetables**				☐	*1 scoop/1 piece*

How often do you consume egg dishes prepared:

		Never/Rarely	Sometimes	Half the time	Mostly	Always
**64a**	**At home**	☐	☐	☐	☐	☐
**64b**	**By food outlet**	☐	☐	☐	☐	☐

Excluding the egg above, how often do you consume one serving of:

		Per day	Per week	Per month	Never/Rarely	One serving means:
**65**	**Egg, fried/scrambled/braised**				☐	*1 egg*
**66**	**Egg, boiled/half-boiled/steamed**				☐	*1 egg*

VEGETABLE DISHES

How often do you consume one serving of:

		Per day	Per week	Per month	Never/Rarely	One serving means:
**67**	**Broccoli**				☐	*1 scoop*
**68**	**Chye sim/mustard greens/xiao bai cai**				☐	*1 scoop*
**69**	**Kailan**				☐	*1 scoop*
**70**	**Kang kong**				☐	*1 scoop*
**71**	**Spinach**				☐	*1 scoop*
**72**	**Cabbage, white**				☐	*1 scoop*
**73**	**Beansprouts**				☐	*1 scoop*
**74**	**Cauliflower**				☐	*1 scoop*

How often do you consume one serving of:

		Per day	Per week	Per month	Never/Rarely	*One serving means:*
75	**Eggplant/Brinjal**				☐	*1 scoop*
76	**Gourd** e.g. bitter, bottle, wax, wintermelon				☐	*1 scoop*
77	**Celery**				☐	*1 scoop*
78	**Ladies fingers/okra**				☐	*1 scoop*
79	**Mushrooms**				☐	*1 scoop*
80	**Carrot**				☐	*1 scoop*
81	**Capsicum/paprika**				☐	*1 scoop*
82	**Dhal/lentils**				☐	*1 scoop*
83	**Peas**				☐	*1 scoop*
84	**Long beans and french beans**				☐	*1 scoop*
85	**Other beans** e.g. kidney bean, baked beans, chick peas				☐	*1 scoop*
86	**Lotus root**				☐	*1 scoop*
87	**Pumpkin**				☐	*1 scoop*
88	**Soy beancurd/tofu/ taukwa/taupok/tempeh**				☐	*1/4 block(3cmx3cm) / 1 piece*
89	**Corn**				☐	*1 cup*
90	**Yam**				☐	*1 slice*
91	**Begedil (potato patty)**				☐	*1 piece*
92	**Potato (inc. mash) (excluding french fries)**				☐	*half a potato/1 cup*

How often do you consume vegetables:

		Never/Rarely	Sometimes	Half the time	Mostly	Always
92a	**In curry with coconut**	☐	☐	☐	☐	☐
92b	**In curry without coconut**	☐	☐	☐	☐	☐
92c	**Stir-fried/pan-fried**	☐	☐	☐	☐	☐
92d	**Deep-fried**	☐	☐	☐	☐	☐
92e	**Stewed/braised**	☐	☐	☐	☐	☐
92f	**Roasted/grilled/baked**	☐	☐	☐	☐	☐
92g	**Boiled/steamed/in soup**	☐	☐	☐	☐	☐

How often do you consume vegetable dishes:

		Never/Rarely	Sometimes	Half the time	Mostly	Always
**92h**	containing **Pork**	☐	☐	☐	☐	☐
**92i**	containing **Chicken**	☐	☐	☐	☐	☐
**92j**	containing **Fish**	☐	☐	☐	☐	☐
**92k**	containing **Beancurd**	☐	☐	☐	☐	☐
**92l**	containing **Other vegetables**	☐	☐	☐	☐	☐

How often do you consume vegetable dishes prepared:

		Never/Rarely	Sometimes	Half the time	Mostly	Always
**92m**	**At home**	☐	☐	☐	☐	☐
**92n**	**By food outlet**	☐	☐	☐	☐	☐

How often do you consume one serving of:

		Per day	Per week	Per month	Never/Rarely	*One serving means:*
**93**	**Fried onion** e.g. onion rings, onion bhaji				☐	*1 piece*
**94**	**Seaweed** e.g. in soup, in Korean/Japanese dishes, as a snack				☐	*3 strips/ 1 dessertspoon*
**95**	**Lettuce**				☐	*4 medium leaves*
**96**	**Coleslaw**				☐	*1 dessertspoon*
**97**	**Raita (yoghurt with cucumber)**				☐	*2 dessertspoons*
**98**	**Cucumber** (excluding in raita)				☐	*3 slices*
**99**	**Tomato, raw**				☐	*half a medium tomato; 5 cherry tomatoes*
**100**	**Preserved vegetables** e.g. kimchi, dong cai, pickled cucumber				☐	*1 dessertspoon*

SALAD DRESSING

How often do you consume one serving of:

		Per day	Per week	Per month	Never/Rarely	*One serving means:*
**101**	**Salad dressing, cream based, low fat** e.g. thousand island, salad cream, mayonnaise				☐	*2 dessertspoons*
**102**	**Salad dressing, cream based, not low fat** e.g. thousand island, salad cream, mayonnaise				☐	*2 dessertspoons*
**103**	**Salad dressing, oil-based** e.g. vinaigrette, olive oil				☐	*2 dessertspoons*

SOUP

How often do you consume one serving of:

		Per day	Per week	Per month	Never/Rarely	*One serving means:*
**104**	**Rasam (tamarind and tomato clear soup)**				☐	*1 medium bowl*
**105**	**Other clear soup** e.g. miso, kimchi soup, broth, stock				☐	*1 medium bowl*
**106**	**Cream soup** e.g. cream of mushroom, cream of tomato				☐	*1 medium bowl*

FRUIT

How often do you consume one serving of:

		Per day	Per week	Per month	Never/Rarely	*One serving means:*
**107**	**Apple**				☐	*1 small*
**108**	**Avocado**				☐	*half a fruit*
**109**	**Banana**				☐	*1 medium*
**110**	**Blueberries**				☐	*1 handful/ 25 blueberries*
**111**	**Dragonfruit**				☐	*half a fruit*
**112**	**Durian**				☐	*1 seed*
**113**	**Grape**				☐	*1 handful/ 15 grapes*
**114**	**Guava**				☐	*half a fruit*
**115**	**Kiwi**				☐	*1 whole*
**116**	**Mango**				☐	*1 slice/ ½ a fruit*
**117**	**Melon** e.g. honeydew, watermelon, rock melon				☐	*1 slice*
**118**	**Orange**				☐	*1 small*
**119**	**Papaya**				☐	*1 slice*
**120**	**Pear**				☐	*1 small*
**121**	**Pineapple**				☐	*1 slice*
**122**	**Strawberries**				☐	*5 strawberries*
**123**	**Dried fruit** e.g. raisins, dates, prunes (excluding in bread/breakfast cereal)				☐	*1 dessertspoon/ 3 dates/prunes*

BEVERAGES

How often do you consume one serving of:

		Per day	Per week	Per month	Never/Rarely	One serving means:
124	**Coffee** (inc. home-made, coffee shop, gourmet e.g. cappuccino)				☐	*1 cup*

What type of milk do you usually add to coffee?

**124a**	**Sweetened condensed milk**	☐
**124b**	**Evaporated milk**	☐
**124c**	**Whole milk/powder**	☐
**124d**	**Low-fat milk/powder**	☐
**124e**	**Skimmed milk/powder**	☐
**124f**	**Soya milk**	☐
**124g**	**Creamer/whitener**	☐
**124h**	**Milk is already added (I drink 3-in-1)**	☐
**124i**	**Milk is already added (I drink 2-in-1)**	☐
**124j**	**Do not add milk**	☐

How much sugar do you usually add to coffee?

**124k**	**Less than 1 tsp**	☐
**124l**	**1-2 tsp/Ask for less**	☐
**124m**	**3-4 tsp/Ask for regular**	☐
**124n**	**5+ tsp/Ask for more**	☐
**124o**	**None**	☐

How often do you consume one serving of:

		Per day	Per week	Per month	Never/Rarely	One serving means:
125	**Tea** (inc. home-made, coffee shop)				☐	*1 cup*

What type of milk do you usually add to tea?

**125a**	**Sweetened condensed milk**	☐
**125b**	**Evaporated milk**	☐
**125c**	**Whole milk/powder**	☐
**125d**	**Low-fat milk/powder**	☐
**125e**	**Skimmed milk/powder**	☐
**125f**	**Soya milk**	☐
**125g**	**Creamer/whitener**	☐
**125h**	**Milk is already added (I drink 3-in-1)**	☐
**125i**	**Milk is already added (I drink 2-in-1)**	☐
**125j**	**Do not add milk**	☐

How much sugar do you usually add to tea?

**125k**	**Less than 1 tsp**	☐
**125l**	**1-2 tsp/Ask for less**	☐
**125m**	**3-4 tsp/Ask for regular**	☐
**125n**	**5+ tsp/Ask for more**	☐
**125o**	**None**	☐

How often do you consume one serving of:

		Per day	Per week	Per month	Never/Rarely	One serving means:
**126**	**Malted drink/hot chocolate** e.g. Milo®, Ovaltine®				☐	*1 cup/can/pack*

What type of milk do you usually add to malted drinks?

**126a**	**Sweetened condensed milk**	☐
**126b**	**Evaporated milk**	☐
**126c**	**Whole milk/powder**	☐
**126d**	**Low-fat milk/powder**	☐
**126e**	**Skimmed milk/powder**	☐
**126f**	**Soya milk**	☐
**126g**	**Creamer/whitener**	☐
**126h**	**Milk is already added (I drink 3-in-1)**	☐
**126i**	**Milk is already added (I drink 2-in-1)**	☐
**126j**	**Do not add milk**	☐

How much sugar do you usually add to malted drinks?

**126k**	**Less than 1 tsp**	☐
**126l**	**1-2 tsp/Ask for less**	☐
**126m**	**3-4 tsp/Ask for regular**	☐
**126n**	**5+ tsp/Ask for more**	☐
**126o**	**None**	☐

Is the malted drink usually:

**126p**	**Packaged**	☐
**126q**	**Freshly prepared/ made up with water**	☐

How often do you consume one serving of:

		Per day	Per week	Per month	Never/Rarely	One serving means:
**127**	**Fruit or vegetable juice (100% juice, no added sugar)** inc. freshly squeezed or pack				☐	*1 glass/bottle/pack*
**128**	**Fruit or vegetable juice drink, not 100% juice** (inc. syrups/cordials with water added) e.g. Ribena, Minute Maid				☐	*1 glass/bottle/pack*
**129**	**Canned/bottled tea** e.g. lemon/peach tea, milk tea, chrysanthemum tea, jasmine green tea				☐	*1 glass/bottle/can*
**130**	**Canned/bottled coffee** e.g. latte, mocha				☐	*1 bottle/can*
**131**	**Other non-carbonated sweetened drinks** e.g. sugarcane juice, bandung, waterchestnut drink, barley drink				☐	*1 glass/bottle/can*
**132**	**Sports drinks** e.g. 100 PLUS®, H-Two-O®				☐	*1 glass/bottle/can*
**133**	**Carbonated soft drinks, sugar free** e.g. Coke Zero®				☐	*1 glass/bottle/can*
**134**	**Carbonated soft drinks, regular** e.g. regular cola, Fanta®				☐	*1 glass/bottle/can*
**135**	**Soya milk, low sugar/no sugar added, inc. flavoured** e.g. chocolate, fruit, red bean				☐	*1 glass/bottle/pack*

How often do you consume one serving of:

		Per day	Per week	Per month	Never/Rarely	*One serving means:*
**136**	**Soya milk, not low sugar, inc. flavoured** e.g. chocolate, fruit, red bean				☐	*1 glass/bottle/pack*
**137**	**Milkshake** e.g. chocolate, strawberry, banana				☐	*1 glass*
**138**	**Yoghurt drink** e.g. lassi, Yakult®, Vitagen®				☐	*1 bottle/glass*
**139**	**Powdered nutrition drink** e.g. whey protein shake, Ensure®, Herbalife®				☐	*1 glass/pack*
**140**	**Alcoholic beverages**				☐	*1 bottle beer; 1 glass wine; 25ml liqueur*
**141**	**Water, plain (inc. bottled)**				☐	*1 glass/bottle*

DAIRY PRODUCTS

How often do you consume one serving of:

		Per day	Per week	Per month	Never/Rarely	*One serving means:*
**142**	**Milk, non-fat (including on cereal but not in tea/coffee)**				☐	*1 glass*
**143**	**Milk, low fat (including on cereal but not in tea/coffee) inc. flavoured** e.g. chocolate, strawberry				☐	*1 glass*
**144**	**Milk, whole (including on cereal but not in tea/coffee) inc. flavoured** e.g. chocolate, strawberry				☐	*1 glass*
**145**	**Yoghurt/frozen yoghurt inc. flavoured** e.g. greek, chocolate, strawberry				☐	*1 pot/pack*

145a *Is the yoghurt usually low fat?* ☐ Yes ☐ No

**146**	**Cheese (inc. cheese spread)**				☐	*1 slice/1 dessertspoon*

146a *Is the cheese usually reduced fat?* ☐ Yes ☐ No

SNACKS AND DESSERTS

How often do you consume one serving of:

		Per day	Per week	Per month	Never/Rarely	*One serving means:*
**147**	**Savoury biscuits** e.g. cream cracker, rice cracker, pretzel				☐	*2 pieces*
**148**	**Sweet biscuits (no filling)** e.g. digestive, marie biscuit, ginger biscuits				☐	*2 pieces*
**149**	**Biscuits/cookies, with filling or with chocolate** e.g. Oreo®, bourbon, Tim Tam®				☐	*2 pieces*
**150**	**Pastry** e.g. egg tart, pineapple tart, red/green bean pastry, croissant, danish, custard puff, cream puff				☐	*1 piece*

How often do you consume one serving of:

		Per day	Per week	Per month	Never/Rarely	*One serving means:*
**151**	**Sweet snack, deep-fried** e.g. pisang goreng, doughnut, you tiao, ham ching peng				☐	*1 piece*
**152**	**Sweet desserts and kueh** e.g. kueh sarlat, kueh talam, kueh ko swee, coconut burfi, putu mayam, ubi kayu, kesari, jelly/agar agar, kuih lapis				☐	*2 pieces*
**153**	**Sweet desserts in soup** e.g. bubur cha cha, pulut hitam, chendol, green bean soup, ice kacang, chng tng, payasam (kheer), grass jelly				☐	*1 bowl*

How often are the sweet desserts and kueh, and the desserts in soup:

		Never/Rarely	Sometimes	Half the time	Mostly	Always
**153a**	**With coconut/coconut milk**	☐	☐	☐	☐	☐

How often do you consume one serving of:

		Per day	Per week	Per month	Never/Rarely	*One serving means:*
**154**	**Beancurd dessert** e.g. tau huay, soya pudding				☐	*1 medium bowl*
**155**	**Pancake/hotcake/waffle** (inc. appam)				☐	*1 piece*
**156**	**Ice cream**				☐	*1 scoop*
**157**	**Cake** e.g. plain butter, sponge, fruit, cream cake				☐	*1 slice*
**158**	**Chocolate**				☐	*1 50g bar*
**159**	**Seeds** e.g. sunflower seeds, sesame seeds				☐	*1 dessertspoon*
**160**	**Nuts** e.g. peanuts, cashews, almonds				☐	*1 dessertspoon*
**161**	**Puffs and pies** e.g. curry puff, chicken pie				☐	*1 piece*
**162**	**Dumpling** e.g. siew mai, wanton, har gow, gyoza				☐	*1 piece*
**163**	**Fried chips and crackers, savoury** e.g. potato chips, prawn crackers, papadam, muruku, keropok, rempeyek, tortilla chips				☐	*1 handful*

DIETARY PRACTICES QUESTIONS

	Per day	Per week	Per month	Never/Rarely
**DP1) How often do you eat at hawker centres, foods courts or coffee shops?**				☐
**DP2) How often do you eat at workplace or school canteens?**				☐
**DP3) How often do you eat at western fast food outlets or quick-service restaurants?**				☐
**DP4) How often do you eat at other restaurants?**				☐
**DP5) How many servings of fruit do you usually eat (excluding juices)?** (1 serving is about 1 apple, 1 banana, 1 papaya wedge)				☐
**DP6) How many servings of vegetables do you usually eat?** (1 serving is about 1 scoop of vegetables)				☐

DP7) What type of tea do you usually consume? (excluding canned/bottled tea) (MAY TICK MULTIPLE OPTIONS)

Chinese tea/oolong tea ☐

English tea/black tea ☐

Green tea ☐

Herbal tea ☐

Do not consume tea regularly ☐

DP8) Do you consume 'less sweet'non-carbonated sweetened drinks, e.g. Nutritea® barley?

Yes ☐

No ☐

DP9) At the table, how often do you add salt or sauces to your food? e.g. soy sauce, braised gravy

Never ☐

Sometimes ☐

Always ☐

DP10) What type of fat or oil is usually used for cooking at home? (TICK ONE ONLY)

Butter, dripping, ghee, lard or any other animal fat ☐

Vanaspati (vegetable ghee) ☐

Vegetable oil/blended oil ☐

Palm oil ☐

Coconut oil ☐

Hard margarine ☐

Soft margarine ☐

Corn oil ☐

Soya bean oil ☐

Sunflower oil ☐

Peanut oil ☐

Canola oil ☐

Olive oil ☐

Others (please specify) ______________________________ ☐

Do not eat food cooked at home ☐

DP11) Do you take vitamins or supplements?

Yes ☐ → Go to Q 11a)

No ☐ → END OF QUESTIONNAIRE

DP11a) If yes, please state the name, brand, number, frequency and duration below.

**Type** e.g. Vitamin C, multivitamin	**Brand name**	**Dose/ Strength** e.g. 500mg (if known)	**Number of pills/ capsules**	**Frequency**			**Length of time taking supplement**		
				**Every day**	**At least once a week**	**Less than once a week**	**Less than 1 year**	**Between 1-5 years**	**More than 5 years**
____________________________	_____________	_______	_______	☐	☐	☐	☐	☐	☐
____________________________	_____________	_______	_______	☐	☐	☐	☐	☐	☐
____________________________	_____________	_______	_______	☐	☐	☐	☐	☐	☐
____________________________	_____________	_______	_______	☐	☐	☐	☐	☐	☐
____________________________	_____________	_______	_______	☐	☐	☐	☐	☐	☐

[END]
