# Supplementary material for: Development of a Semi-Quantitative Food Frequency Questionnaire to Assess the Dietary Intake of a Multi-Ethnic Urban Asian Population
Source: Nutrients. 2016 Aug 27;8(9):528. doi: 10.3390/nu8090528 (PMC5037515; doi:10.3390/nu8090528)
Supplement: Supplementary file 1 [file nutrients-08-00528-s001.zip › nutrients-142106-supplementary-publish/Suppl_materials/online supplementary materials.docx]

Supplementary Materials: Development of a Semi-Quantitative Food Frequency Questionnaire to Assess the Dietary Intake of a Multi-Ethnic Urban Asian Population

Nithya Neelakantan, Clare Whitton, Sharna Seah, Hiromi Koh, Salome A. Rebello, Jia Yi Lim, Shiqi Chen, Mei Fen Chan, Ling Chew and Rob M. van Dam

**Table S1.** Foods that explained at least 60% of between-person variation in total energy intake in the 24-h dietary recall survey by ethnicity.

| **Chinese** | | **Malay** | | **Indian** | |
| --- | --- | --- | --- | --- | --- |
| Food group | Cumulative *R*^2^ * | Food group | Cumulative *R*^2^ * | Food group | Cumulative *R*^2^ * |
| Pastries | 0.10 | White rice | 0.12 | Grapes | 0.15 |
| White rice | 0.20 | Garlic spread | 0.22 | White rice | 0.25 |
| Tea | 0.26 | Flavoured rice | 0.32 | Gravy with coconut | 0.33 |
| French fries | 0.31 | Non-carbonated sweetened drinks ^2^ | 0.37 | Fried rice, plain or with meat/fish | 0.39 |
| Nuts | 0.35 | Puffs and pies | 0.41 | Pasta with meat/fish/vegetables | 0.45 |
| Puffs and pies | 0.39 | French fries | 0.44 | French fries | 0.50 |
| Fried noodles | 0.42 | Fried noodles | 0.47 | Dhal ^3^ | 0.55 |
| Red/brown rice | 0.46 | Oranges | 0.50 | Non-carbonated sweetened drinks ^2^ | 0.58 |
| Hazelnut and chocolate-flavoured spread | 0.49 | Nuts | 0.53 | Puffs and pies | 0.61 |
| Okra dishes | 0.51 | Fried chips and crackers, savoury | 0.55 | Chapati ^4^ | 0.64 |
| Prawn/crustaceans dishes | 0.53 | Pancake/hotcake/waffle | 0.57 | Fried noodles | 0.67 |
| Melon | 0.55 | Beef dishes | 0.59 | Chicken dishes | 0.69 |
| Egg, fried/scrambled/braised | 0.57 | Eggplant dishes | 0.61 | Tea | 0.70 |
| Egg-based mixed dishes ^1^ | 0.59 | Dumplings | 0.63 | Powdered nutrition drinks | 0.72 |
| Coffee | 0.61 | Red/brown rice | 0.64 | Soybean curd dishes | 0.74 |

* Values are adjusted *R*^2^ using forward regression rather than stepwise in order to assign an *R*^2^ value to each food group. The ordering shows the process and in the final model, values may no longer be ordered according to size since addition of later items may reduce or increase values of earlier items. ^1^ e.g., vegetable omelette; ^2^ excluding juices and bottled tea/coffee; ^3^ lentil stew; ^4^ whole wheat Indian bread.

**Table S2.** Top 20 contributors according to percent contribution to total fiber intake in the 24-h dietary recall study by ethnicity.

|  | **Chinese (*n* = 318)** |  |  | **Malay (*n* = 244)** |  |  | **Indian (*n* = 243)** |  |  |
| --- | --- | --- | --- | --- | --- | --- | --- | --- | --- |
| Rank | Food items | % of fibre | % consumers | Food items | % of fibre | % consumers | Food items | % of fibre | % consumers |
| 1 | Fried noodles | 7.05 | 42 | White bread | 7.29 | 55 | Dhal ^2^ | 9.22 | 47 |
| 2 | White rice | 5.43 | 81 | White rice | 6.46 | 88 | White bread | 5.63 | 53 |
| 3 | Noodles in soup | 5.29 | 37 | Chicken dishes | 6.04 | 64 | White rice | 4.97 | 86 |
| 4 | White bread | 4.15 | 42 | Fried noodles | 4.25 | 30 | Fried noodles | 4.18 | 30 |
| 5 | Dry noodles | 3.48 | 22 | Flavoured rice | 3.69 | 34 | Wholemeal bread | 4.10 | 23 |
| 6 | Apples | 3.17 | 27 | White fish dishes | 3.29 | 44 | Chapati ^3^ | 3.25 | 17 |
| 7 | Flavoured rice | 3.09 | 23 | Puffs and pies | 2.77 | 21 | Thosai ^4^ | 3.00 | 25 |
| 8 | Pears | 2.78 | 16 | Oily fish dishes | 2.77 | 43 | Apples | 2.69 | 30 |
| 9 | Wholemeal bread | 2.71 | 15 | Potato dishes | 2.31 | 20 | Flavoured rice | 2.47 | 23 |
| 10 | Cabbage dishes | 2.04 | 38 | Apples | 2.27 | 19 | Potato dishes | 2.41 | 38 |
| 11 | Chicken dishes | 1.81 | 60 | Wholemeal bread | 2.25 | 14 | Chicken dishes | 2.12 | 44 |
| 12 | Papaya | 1.72 | 11 | Noodles in gravy | 1.99 | 10 | White fish dishes | 1.77 | 26 |
| 13 | White fish dishes | 1.67 | 47 | Beef dishes | 1.93 | 25 | Papaya | 1.69 | 13 |
| 14 | Banana | 1.65 | 14 | Roti prata ^1^ | 1.76 | 20 | Mutton and lamb dishes | 1.62 | 12 |
| 15 | Filled buns, savoury | 1.63 | 13 | Malted drinks | 1.74 | 30 | Idli ^5^ | 1.62 | 11 |
| 16 | Nuts | 1.58 | 5 | Banana | 1.63 | 15 | Bean dishes | 1.58 | 27 |
| 17 | Pork dishes | 1.57 | 50 | Cabbage dishes | 1.50 | 23 | Pears | 1.56 | 7 |
| 18 | Potato dishes | 1.38 | 17 | French fries | 1.42 | 14 | Carrot dishes | 1.47 | 35 |
| 19 | Sweet desserts in soup | 1.32 | 7 | Dry noodles | 1.42 | 6 | Roti prata | 1.39 | 16 |
| 20 | Bean dishes | 1.32 | 16 | Soybean curd dishes | 1.35 | 21 | Fried chips and crackers, savoury | 1.37 | 21 |

^1^ flour based pancake; ^2^ lentil stew; ^3^ whole wheat Indian bread; ^4^ fermented rice pancake/crepe; ^5^ savoury steamed rice cake.
